# Considering Proximal Urea Cycle Disorders in Expanded Newborn Screening

**DOI:** 10.3390/ijns6040077

**Published:** 2020-10-08

**Authors:** Tania Vasquez-Loarte, John D. Thompson, J. Lawrence Merritt

**Affiliations:** 1 Public Health Genetics, University of Washington, Seattle, WA 98195, USA; taniavasquezloarte@gmail.com; 2 Newborn Screening Laboratory, Public Health Laboratories, Washington State Department of Health, Shoreline, WA 98155, USA; john.thompson@doh.wa.gov; 3 Department of Pediatrics, University of Washington, Seattle, WA 98105, USA

**Keywords:** proximal urea cycle disorders, ornithine transcarbamylase deficiency, carbamoyl phosphate synthetase 1 deficiency, N-acetyl glutamate synthetase deficiency, neonatal screening, public health, newborn screening, NBS

## Abstract

Proximal urea cycle disorders (PUCDs) have adverse outcomes such as intellectual disability and death, which may benefit from newborn screening (NBS) through early detection and prevention with early treatment. Ornithine transcarbamylase deficiency (OTCD) and carbamoyl phosphate synthetase 1 deficiency (CPS1D) are screened in six and eight states in the United States. We analyzed current evidence to see if it supports inclusion of PUCDs in the NBS panels based upon prevention potential, medical, diagnostic, treatment, and public health rationales. A literature review was performed in PubMed using MESH terms for OTCD, CPS1D, and NAGSD. A systematic review was performed in the hallmark of NBS inclusion criteria. We reviewed 31 articles. Molecular and biochemical diagnosis is available to provide diagnostic evidence. Untreated PUCDs have a significant burden with considerable developmental delay and mortality that may improve with early treatment. Tandem mass spectrometry can be used for NBS for PUCDs; however, citrulline and glutamine alone are not specific. Medical treatments currently available for PUCDs meet existing medical, diagnostic, treatment, and public health rationales. Improvement in NBS algorithms to increase sensitivity and specificity will allow earlier diagnosis and treatment to potentially improve disability and mortality rates.

## 1. Introduction

Newborn screening (NBS) is a population-based, preventive public health approach aimed at the early identification and treatment of certain diseases that otherwise are lethal or chronic and disabling [1]. The United States Department of Health and Human Services provides a Recommended Uniform Screening Panel (RUSP); however, each state has individual decision-making for NBS panels.

In Washington State, the Board of Health oversees which diseases are included in the NBS panel in compliance with the Revised Code of Washington (RCW 70.83.050). In this way, it nominates an Advisory Committee for the evaluation of each candidate condition. This evaluation is guided by three main principles: (1) the decision should be supported by evidence; (2) there should be universal accessibility to diagnostic and therapeutic services; and (3) the advantages of screening for a disease should exceed the potential harms to children, their families, and society. Likewise, Washington State has outlined criteria for inclusion of any condition in the NBS panel that address core criteria to guide implementation of NBS for a condition [2].

1. Public Health Rationale
2. Diagnostic Testing Rationale
3. Prevention Potential and Treatment Rationale
4. Newborn Screening Rationale [2]

The fifth NBS criterion is cost-benefit/cost-effectiveness and will not be considered in this analysis. Currently the Washington State NBS panel includes 34 conditions screened for in approximately 83,000 newborns per year through two routine screens with estimates of prevention of death or disability in 150–200 babies/year [3]. Washington State performs two NBS tests routinely. The first sample is collected typically between the first 18 to 48 h of life and the second sample within the 7th and 14th day of life [4]. Positive results on the first screen are typically reported to primary care providers five to six days after birth (unpublished data from Washington NBS). 

Urea cycle disorders (UCDs) are a consequence of defects in the enzymes and the transporters of the urea cycle, which is the key pathway in elimination of waste nitrogen as ammonia into urea. For the purpose of this manuscript, we will focus on proximal urea cycle disorders—in fact, 40% of UCD infants die in the newborn period and 52% have developmental delay at one year of age [5]. Similar to the RUSP, and most states and nations, the only UCDs included in the Washington State NBS panel are the distal UCDs: argininosuccinate synthetase deficiency (ASSD, also called citrullinemia type I), and argininosuccinate lyase deficiency (ASLD, also called argininosuccinic acidemia) [1,4]. Only a few states include the proximal UCDs (PUCD): ornithine transcarbamylase deficiency (OTCD)—the most common UCD—carbamoyl phosphate synthetase 1 deficiency (CPS1D), and N-acetylglutamate synthetase deficiency (NAGSD). PUCDs frequently present in the newborn period and have significant morbidity and mortality. Critics question if implementation of NBS in PUCDs will improve adverse outcomes [6], as evidence is unclear if NBS may have any benefit in both early onset (EO, ≤30 days of life) and late onset (LO, >30 days of life) presentations. Furthermore, it is questionable whether results will be available early enough to prevent disability or death in all children with EO PUCDS (note to reader: we use 30 days for this review as our best attempt to provide a definition for early vs. late onset presentations, noting this is variable and not always clearly defined in many manuscripts, making analysis difficult).

We report our findings from a retrospective review of currently available literature relating to PUCDs and their inclusion in NBS—using the principles and rationale from Washington State as proof of concept.

## 2. Methods

To elaborate this narrative review, we performed a literature search in PubMed and covered publications from 1990 to April 2020. We used Medical Subject Headings for OTCD, CPS1D, NAGSD, analysis, blood, complications, diagnosis, diet therapy, drug therapy, epidemiology, etiology, genetics, metabolism, mortality, statistics and numerical data, therapy, urine, and screen. We included observational studies, systematic reviews, randomized controlled trials, and real-world evidence in English and Spanish. Because we anticipated that outcomes data would be more informative to assess the criteria outlined by the NBS Program, we excluded case reports and pre-clinical studies. Publications meeting inclusion criteria were reviewed and analyzed in the hallmark of the inclusion criteria of Washington State [2]. Even though this is not a systematic review, efforts were made to follow the PRISMA guidelines (Table S1) [7]. The review of the publications retrieved was performed by two of the authors. Results regarding the therapeutic and screening rationale were summarized in Tables. For the public health rationale, we show the results about incidence data, proportions of neonatal onset, developmental delay and mortality reported in Burgard et al. 2016 and Summar et al. 2008, which are the most complete to our knowledge [5,8]. 

## 3. Results 

A total of 404 results were identified; however, 10 were duplicate publications and 360 met exclusion criteria (Figure S1). A list of the included 31 references are summarized in Table S2. 

### 3.1. Public Health Rationale

The literature was reviewed justifying the current status of population-based vs. risk-based screening. According to international registries analyzing NBS data, the prevalence of all UCDs is approximately <1:35,000 in North America. OTCD is the most common PUCD having an approximate incidence of 1:56,500 (Table S3), making it similar in order, or more common, than many other commonly accepted distal UCDs and non-UCD NBS conditions currently in many NBS panels (e.g., very long-chain acyl-coenzyme A dehydrogenase deficiency [9], systemic primary carnitine deficiency [10], methylmalonic acidemia *MMUT* [11], propionic academia [12]) [13]. Meanwhile, the incidence of CPS1D and NAGSD is likely less than 1:1,000,000—making these ultra-rare conditions.

Early presentations are common in CPS1D and OTCD [5]. Among these EO patients, 60% survive the neonatal period and about 19% of them die by the end of the first year of life. Among the patients who survive after the first year of life, 47–67% have developmental delay, and 15–20% have a normal development (Table 1) [5,14]. 

Furthermore, even though OTCD is an X-linked recessive disorder, two studies report 19% and 22% of symptomatic newborns with EO OTCD are female, higher than historical estimates [15,16]. One study reports 79% of OTCD males and 96% of OTCD females may remain asymptomatic at 6 days of life, theoretically enabling NBS to prevent first symptoms [17]. 

The epidemiology of LO UCDs is less clear due to nonspecific symptoms and missed diagnoses, but it has been estimated to affect approximately 50% of PUCDs [18,19]. Lower mortality and developmental delay rates are seen in LO presentations (Table 2) [16,20]. OTCD provides the best available information on LO presentations, being more common (70%–78%) and primarily diagnosed between 1 month and 16 years old (82.5%), with ammonia and glutamine levels ranging between 60 to 500 µmol/L and from 570 to 8175 µmol/L, respectively (Brassier 2015). Females are most frequently affected in the LO group (60%–71%) [15,16].

Diagnostic delays can be significant—ranging from 1 day up to 1134 days for patients with EO OTCD and EO CPS1D, even to extremely large times of 3652 days in a child with NAGSD—when depending upon development of disease symptoms to initially identify disease [27]. 

Recurrence of hyperammonemic episodes is high in patients with EO and LO PUCDs [18,19]. For patients with ASSD, OTCD, or CPS1D, 70% of episodes occur between 31 days to 12 years of life with a frequency of 2.4–2.9 episodes per year, which can be as high as eight episodes within the first year of age for a patient with CPS1D. For example, one female with OTCD has been reported with 77 episodes during her lifetime (average 3.5 events/year between 2 and 23 years old) [8]. Benefits of early detection and early treatment are seen within the first year of life, as well as in LO PUCD; what contribution NBS may have to this remains to be proven [18]. Odds ratios trended towards lower odds for movement disorder and delayed milestones in distal UCDs in the early diagnosis groups and NBS compared to later groups detected through clinical symptoms [17].

### 3.2. Diagnostic Testing Rationale

Diagnostic testing should be accurate, be done with expertise, and be available for infants identified with a possible PUCD [2]. Diagnostic testing is currently available in many clinical chemistry, biochemical, and molecular genetic laboratories around the world [6]. The most significant current challenges to diagnostic testing based on clinical presentations often relates to poor awareness and recognition of UCD-associated signs and symptoms and then ordering appropriate diagnostic testing through ammonia levels, quantification of plasma amino acids, and urinary orotic acid. Elevations of ammonia and glutamine, decreased levels of citrulline and arginine, and other suggestive amino acid abnormalities and routine chemistries reflecting liver disruption (e.g., AST, ALT) and function (e.g., prothrombin activity, albumin) may increase suspicion of an underlying PUCD [6,16]. 

Diagnostic confirmation through genetic testing is necessary for an infant with a suspected PUCD. Biochemical abnormalities on plasma and urine testing may not separate 100% of potential PUCDs following an abnormal NBS [28]. Genetic testing is available in clinical laboratories for suspected PUCD patients and will also be helpful for recurrence-risk assessments for carrier identification, prenatal diagnosis, and genetic counseling [15,16]. Currently molecular sequencing and/or multiplex ligation-dependent probe amplification for deletion and duplication detection will identify most CPS1D and NAGSD but is limited to approximately 80% of the mutations in OTCD [15,16,29]. 

Measurement of enzyme activity from liver or intestinal mucosa biopsy is recommended if the metabolite pattern or genetic testing is non-informative or inconclusive or may be reserved for research [30]. While molecular confirmation may not be possible with current technology for less than 20% of OTCD patients [6,29], ongoing research into deep genomic sequencing may resolve this. 

### 3.3. Prevention Potential and Treatment Rationale

Implementing timely treatment in PUCDs is considered crucial to preventing adverse outcomes [2,15]. Recommendations for implementation of acute and chronic treatments, regardless of the age of onset are available [6,30]. Treatment modalities consists of: (1) dietary protein restriction; (2) prevention of catabolism; (3) use of nitrogen scavengers; (4) use of citrulline and arginine amino acid supplementation; (5) hemofiltration; and (6) liver transplantation [8,26]. Measuring therapeutic outcomes is challenging due to a lack of randomized controlled trials; most data come from non-randomized, uncontrolled open label studies and follow varying treatment guidelines, treatment availability, registration procedures, different follow-up periods, health systems, clinical awareness, ethnic background, etc. Furthermore, patients often receive more than one treatment at a time, so attributing specific benefits and outcomes is very difficult. The increased duration of coma and increased ammonia level (e.g., peak or total exposure such as area under the curve) during crisis are thought to contribute to increased morbidity and mortality [15]. The use of nitrogen-scavengers or liver transplant has demonstrated a 79%, 92.8%, and 96% decrease in the post-treatment ammonia levels compared to baseline, respectively (Table 2) [21,23,25]. 

To date, there are no studies that evaluate the effect of current therapies on mortality. There is an apparent decrease in mortality rates if we descriptively compare this outcome in studies that report the use of scavengers and dialysis and transplant. The comparison of different studies published in different timeframes show differences in outcomes. Focusing our review upon OTCD, one early study of EO OTCD between 1971–2011 reports a mortality of 74% during the neonatal period or follow-up [15]. More recent studies from the periods 1980–2005 [21], 2001–2013 [26], and 2012–2013 [16], report mortality rates of 47%, 43%, and 40% for patients with EO OTCD, respectively. Similarly, in LO OTCD, in two studies published in 2005 and 2015, the mortality and developmental delay rates decreased from 34% to 13% and from 37% to 29%, respectively [15,18]. Interestingly, among LO OTCD patients who have developmental delay, 70% are women [18]. Comparing two studies from Japan, the survival in LO OTCD increased from 42% (study period 1978–1985) to 92% (1999–2009), with 23% of these last group having access to liver transplant [22,31].

Hemofiltration may be initiated in patients with hyperammonemia resistant to other treatments and is very effective in decreasing ammonia levels. The removal of ammonia through hemofiltration has been proposed when ammonia levels are >180, >200, >500 µmol/L, or if ammonia levels do not adequately decrease within 3–6 h after the start of therapy [6,14,22]. In a review of 90 published case reports on outcomes in patients with UCD who received dialysis there was a decrease in the mortality rate from 50% (1971–1990) to 20.8% (2011–2016). However, they conclude hemodialysis does not influence the patients’ outcome [14]. This should be carefully analyzed as higher ammonia levels are related to worse clinical outcomes (disability and death) and dialysis would be an intermediate variable. In fact, in this cohort, deceased patients had higher trigger ammonia levels compared to survivors (1501 µmol/L ±1052 vs. 1097 µmol/L ±762) [14]. Interestingly, two studies reported zero deaths with lower dialysis trigger ammonia levels of ≤359 µmol/L and <180 µmol/L [14,20]. Therefore, it is reasonable to consider lowering the trigger ammonia levels for hemofiltration to potentially reduce the risk of disability and death [6,30]. 

Liver transplantation does not reverse neurologic compromise; however, it normalizes ammonia levels and eliminates the need for dietary restrictions or nitrogen-scavenging medications. Therefore, transplant is recommended before neurodevelopmental compromise occurs [24]. Whole deceased donor liver transplant (DDLT), partial DDLT, and living donor transplant have similar survival rates [23,24]. There is a decrease in mortality rate in UCD patients who received liver transplants after a 5-year follow-up period (10% vs. 0% when comparing before 2004 vs. 2001–2012, respectively), which may reflect new management of liver transplantation overall [23,24]. Furthermore, delayed liver transplant is associated with cognitive impairment regardless of the disease of onset, however the study does not recommend a specific timeframe [23,32]. A recent guideline recommends liver transplant should be performed in patients with recurrent decompensations despite standard medical treatment, ideally 3–12 months of age, and when body weight is >5 kg to obtain a favorable outcome [6]. 

### 3.4. Newborn Screening Rationale

**Figure 1:**
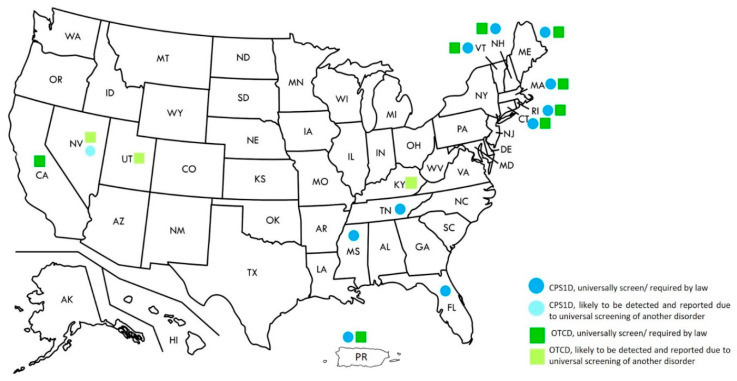
Newborn screening for Urea Cycle Disorders in the United States, 2020.

While NBS for distal UCDs are recommended in the United States’ RUSP, these are more variable in other countries [1,17]. Currently, screening for CPS1D and OTCD is required by law in ten and eight states, respectively. Screening for NAGSD has not been specifically mentioned in any state; however, screening for OTCD and CPS1D will also automatically detect patients with NAGSD (Figure 1) [33].

NBS for UCDs is performed by tandem mass spectrometry (MS/MS) [28,34]. Glutamine and citrulline are commonly considered the primary markers to screen for PUCDs, but because of their variability, a screening tool that combines multiple markers may be helpful. Because glutamine is unstable, to avoid a 5% decrease in concentration, dried blood spots should be analyzed within 2 weeks after collection [34]. Low citrulline concentrations are non-specific and can be found in newborns who are protein restricted, those with intestinal pathologies, mitochondrial disorders, premature infants, and other inborn errors of metabolism [6,28,30,34,35]. The measurement of orotic acid in dried blood spots using MS/MS has been validated previously, and showed results significantly different between samples from healthy newborns and those affected by OTCD in stand-alone and multiplex methods [36,37]. Elevated levels are also seen in ASS, ASL, infections and other physiologic conditions such as nutritional status. Age at specimen collection may alter orotic acid concentration. 

Alternative strategies for NBS are being developed. The Region 4 Stork Project, which was an international collaboration in the US and 45 other countries, released the results of a study that aimed to achieve the clinical validation of cutoff values for NBS by MS/MS. This study included participants with CPS1D and OTCD (*n* = 60) and used CPS1D and OTCD markers such as citrulline to arginine, citrulline to phenylalanine, glutamine/citrulline, glutamic acid/citrulline, and methionine/citrulline ratios [38]. An exploratory pilot study tested a state specific Region 4 Stork tool on 11 patients with PUCDs using the concentration of citrulline, arginine, alanine, methionine, phenylalanine, glutamine, and various ratios. This tool detected known EO and LO PUCD patients except for one asymptomatic LO OTC patient and a second patient with an incomplete set of analytes—both had normal citrulline levels [39]. 

Published prospective outcomes from NBS programs including OTCD, CPS1D, and NAGSD are rare, so it is difficult to evaluate if NBS has any impact upon decreasing longer term mortality or preventing developmental delay, especially in LO patients. For instance, in a European UCD cohort of 10 patients identified by NBS, only one of them had OTCD [40]. The NBS program in Singapore published eight years of experience screening 177,267 infants, detecting four false positive cases and failing to detect one case of OTCD [41].

Retrospective studies have attempted to provide information about the impact of NBS for PUCDs. A retrospective study reported only three patients with PUCD identified via NBS, preventing any significant assessment of outcomes compared to symptomatic diagnosis; although positive trends were seen when looking at all UCD patients [17]. An analysis of ASS1D and ASLD identified through NBS showed an apparent positive effect on cognitive measures; although further study is needed measure any effect from “mild or benign” mutations being detected by NBS [32]. Further studies with larger populations are necessary to evaluate the benefit in reducing the diagnostic odyssey in PUCDs. 

## 4. Discussion

In this review we found that the literature provides support for adding PUCDs to NBS based on current medical, diagnostic, therapeutic, and public health rationales. The complications of PUCDs are disabling or lethal as 11% will die within the first year of life and 31% will have developmental delay [5]. Outcomes are further aggravated by delayed diagnoses [19], and potentially unnecessary procedures and treatments. For every male and female child diagnosed with OTCD, we would expect 2.9 and 2.4 recurrence episodes per year, respectively [8], which lead to hospitalizations and intensive care treatment [42]. The implementation of NBS for EO CPS1D, OTCD, and NAGSD provides the potential for implementing earlier treatment, thus potentially preventing some of the mortality and developmental delay in these patients that occur despite current guidelines [18,19]. Reducing the age of diagnosis though NBS theoretically could diagnose up to two thirds of all UCD patients, or about 70% of male OTCD and 95% of female OTCD, at day 12 of life while they still remain asymptomatic [17]. 

Advances in hemodialysis and liver transplant, and better established patient care protocols and guidelines have improved survival and developmental outcomes in children with EO and LO PUCDs [5,14,23,24]. However, further specific data are needed to stratify these outcomes by type, age of diagnosis, genotype, disease severity, and delays in implementing treatment. The development of individualized genetic therapies is necessary for both EO and LO PUCDs.

Although there are concerns with the effectiveness of current screening algorithms, NBS is still a promising population-based approach that aims to decrease the rates of undiagnosed patients, developmental delay, and mortality in newborns. While current guidelines support prenatal screening as an optimal screening method [6,42,43], this only targets at-risk populations with positive family history, neglecting early and later-onset affected patients with no prior or a poor documented family history. The impact of OTCD diagnosis from NBS will certainly lead to other family members being diagnosed, further expanding our understanding of the clinical spectrum of OTCD. A common concern is that symptoms of PUCDs frequently develop at less than 6 days of life—before NBS results are typically available. Acknowledging the limited benefit in this very early group and noting a prospective trail may be helpful, growing evidence supports benefit and improved health outcomes in at least the 48% of patients who survive the first year of life [5]. Additionally, further study of the detection rate of OTCD carriers and LO PUCDs by NBS is needed. One retrospective study found 52.4% of UCD patients diagnosed in the first 10 days of life remained asymptomatic including 24.5% of female OTCD and 15.1% of male OTCD. Together with LO UCD who develop symptoms between 10–28 days of life, nearly 61% of UCD patients may be pre-symptomatically diagnosed by NBS [27]. Some LO OTCD patients were detected in the pilot study by Merritt et al., showing proof of concept, but a larger population based study is clearly needed; of note, a routine second NBS as performed in WA state may be helpful in these studies [39]. Specific data supporting any impact of pre-symptomatic diagnosis in PUCD patients are needed, while evidence of improved outcomes following NBS with ASSD and ASLD continues to grow [17,27,32]. Theoretically, EO PUCD patients presenting after the first week of life and all LO PUCD patients, may also have similar benefit from NBS.

Continued validation of NBS methodologies is needed. Improvements in amino acid quantification by MS/MS, in implementation of multivariant tools, or in alternative amino acid ratios need further development for increasing sensitivity, specificity, and positive predictive values in larger populations. We will continue to benefit from the experience of states currently screening for OTCD and CPS1D in screening methods, tools, and follow-up protocols used to address the significant concern of false positives. Designing proactive strategies to create appropriate confirmatory protocols and to educate both health professionals and the general public may mitigate parental anxiety secondary to false positives results [44].

The absence of published cost-effectiveness analyses is a limitation of this study. For this purpose, collaborative projects with NBS programs that currently include PUCDs are needed to provide cost estimates related to the diagnostic procedures and indirect costs that finding true positive cases entails. The limited number of clinical trials to determine the effectiveness and safety of the available treatments is another limitation to this study. However, to address this limitation, we included evidence from observational studies or cohorts from different research networks and countries. Finally, the conclusions from this study are applicable to countries or regions that have a similar access to NBS technologies and therapeutics. In countries with limited resources, we recommend evaluating their accessibility to NBS technologies, diagnostic methods, and therapeutics prior to implementing NBS. Additional steps such the development of screening criteria, guidelines for the diagnosis and management of PUCDs, and the training of human resources are also recommended in these settings.

## 5. Conclusions

NBS is a public heath responsibility aiming to prevent death or disability and optimize outcomes. NBS for PUCD might benefit newborns presenting later than the first few days of life. While PUCDs meet the medical, diagnosis, treatment, and public health rationales, ongoing work to improve the effectiveness of the screening test will make a stronger case for NBS for PUCDs as an effective screening test continues to be necessary.

## Figures and Tables

**Table 1:** Burden of disease for patients with early onset Urea cycle disorders (UCDs) (Adapted from Burgard 2015) [5].

Disorder	Proportion (PR) of Early Onset		Outcome of Early Onset Patients Who Survived to 1 Year of Age			
	*n*	PR (95%CI)	*n*	Mortality PR (95% CI)	Developmental Delay PR (95% CI)	Normal PR (95% CI)
CPS1D	148	0.75 (0.61–0.88)	23	0.34 (0.18–0.54)	0.47 (0.3–0.68)	0.20 (0.07–0.38)
OTCD Males	517	0.52 (0.39–0.65)	44	0.18 (0.01–0.44)	0.67 (0.35–0.88)	0.15 (0–0.39)
OTCD Females	434	0.07 (0.03–0.11)	3	n/a	n/a	n/a

CI = 95th percentile confidence interval.

**Table 2:** Outcomes in proximal urea cycle disorders and ornithine transcarbamylase deficiency (OTCD).

Year (Study Period)	Participants	Treatment	Mortality	Mortality for OTCD	Developmental Delay
**2007** (1980–2005) [21]	299 patients, EO 31.2%, LO 68.8%, OTCD 654	SB, SP, D 35%	EO 27 % LO 6%,	After 1st episode: OTCD EO 39.5% OTCD LO 8%;	N/A
**2008** (1982–2003) [8]	260 patients, EO 34%, LO 66%, PUCD 178, DUCD 79, OTCD 142,	SB, SPB	Overall: 65% EO 32% LO 10–20% PUCD 26–47% DUCD 22%	OTCDm 47% OTCDf 26%	N/A
**2012** (1999–2009) [22]	177 patients, EO 43.5%, LO 51.4%, 139 PUCD, 38 DUCD, 57 OTCD	AA, SC, HF, LT.	EO 16% LO 10% PUCD 12% DUCD 10%	OTCD EO (5 y) 14% OTCD LO (5 y) 8%	LT patients: 14% of those with peak ammonia between 60–180 µmol/l and in 51% of those with peak ammonia >360 µmol/L
**2013** (2001–2012) [23]	23 patients, 13 PUCD, 10 DUCD, 8 OTCD	LT	0% over 5 years	N/A	48% (similar to pretransplant) PUCD 60% (6/10) DUCD 46% (6/13)
**2014** (2012–2013) [16]	104 patients, EO 25.9%, LO 61.5%, 70 PUCD, 34 DUCD, 67 OTCD	AA, SC	3%	OTCD EO 40% (2/5) OTCD LO 0%	OTCD EO unknown OTCD LO 48 % DUCD 61% (13/21)
**2015** (1971–2011) [15]	90 OTCD EO 30%, LO 70%	AA, SC, D	N/A	OTCD EO 74% (60% at 1st episode) OTCD LO 13%	“EO and LO had similar neurological scores”
**2015** (1999–2003) [24]	21 patients, 6 OTCD	LT	5 y: 14.3%	OTCD 17% (unrelated to LT)	N/A
**2016** (2000–2010) [25]	61 patients, EO 40.9%, LO 59.01%	AA, SC, HF 2.4%	13% (8/61 all EO)	N/A	N/A
**2016** (2001–2013) [26]	63 (all EO) 35 PUCD, 27 DUCD, 23 OTCD,	AA, SC, LT 17%	PUCD (5 y) 40% DUCD (5 y) 14.8% Neonatal, all: 25.4%	OTCD (5 y) 43%	N/A
**2018** (1971–2016) [14]	202 EO, 118 PUCD, 84 DUCD, 66 OTCD	AA, SC, D 71%	PUCD: 33%	OTCD 36.3%	Overall 80% PUCDs 37.3% DUCDs 34.5%
**2018** (1999–2009) [20]	177 patients, 116 OTCD	LT 23.7%	PUCDs 11%	OTCD EO 14% OTCDm LO 10% OTCDf LO 12%	OTCD LO 21% OTCDm LO 23% OTCDf LO 20%

EO—Early onset; LO—Late onset; OTCD—ornithine transcarbamylase deficiency; OTCDm—males affected with ornithine transcarbamylase deficiency; OTCDf—females affected with ornithine transcarbamylase deficiency; LT—liver transplant; SB—sodium benzoate; SP—sodium phenylacetate; SPB—sodium phenylbutyrate; HF—hemofiltration; HD—hemodialysis; D—dialysis; AA—amino acids; SC—scavengers; PUCD—proximal urea cycle disorders; DUCD—distal urea cycle disorders; 5 y—5 year follow-up.

## Supplementary Materials

The following are available online at https://www.mdpi.com/2409-515X/6/4/77/s1, Figure S1. Selection criteria for articles included in the study. Table S1. Prisma Checklist. Table S2. Summary of Literature Included in Systematic Review.

## References

[1] Health Resources & Services Administration Recommended Uniform Screening Panel. https://www.hrsa.gov/advisory-committees/heritable-disorders/rusp/index.html.

[2] Washington State Board of Health Washington State Board of Health Process to Evaluate Conditions for Inclusion in the Required Newborn Screening Panel. https://sboh.wa.gov/Portals/7/Doc/HealthyBehaviors/NBS/NewbornScreeningCriteria_Updated_12032015_KK.pdf.

[3] Nucup-Villaruz C S.S. The Compelling Benefits of Routine 2nd NBS: A Fifteen-Year Review in Washington State. https://www.aphl.org/conferences/proceedings/Documents/2014/NBS/37Nucup-Villaruz.pdf.

[4] Washington State Board of Health Healthcare Provider Manual: The Complete Guide to Newborn Screening in Washington State. https://www.doh.wa.gov/Portals/1/Documents/5220/ProviderManual.pdf.

[5] Burgard P., Kölker S., Haege G., Lindner M., Hoffmann G.F. (2016). Neonatal mortality and outcome at the end of the first year of life in early onset urea cycle disorders—Review and meta-analysis of observational studies published over more than 35 years. J. Inherit. Metab. Dis..

[6] Häberle J., Burlina A., Chakrapani A., Dixon M., Karall D., Lindner M., Mandel H., Martinelli D., Pintos-Morell G., Santer R. (2019). Suggested guidelines for the diagnosis and management of urea cycle disorders: First revision. J. Inherit. Metab. Dis..

[7] Moher D., Shamseer L., Clarke M., Ghersi D., Liberati A., Petticrew M., Shekelle P., Stewart L.A., Group P.-P. (2015). Preferred reporting items for systematic review and meta-analysis protocols (PRISMA-P) 2015 statement. Syst. Rev..

[8] Summar M.L., Dobbelaere D., Brusilow S., Lee B. (2008). Diagnosis, symptoms, frequency and mortality of 260 patients with urea cycle disorders from a 21-year, multicentre study of acute hyperammonaemic episodes. Acta. Paediatr..

[9] Merritt J.L., Norris M., Kanungo S. (2018). Fatty acid oxidation disorders. Ann. Transl. Med..

[10] El-Hattab A.W., Adam M.P., Ardinger H.H., Pagon R.A., Wallace S.E., Bean L.J.H., Stephens K., Amemiya A. (1993). Systemic Primary Carnitine Deficiency. GeneReviews^®^.

[11] Almasi T., Guey L.T., Lukacs C., Csetneki K., Voko Z., Zelei T. (2019). Systematic literature review and meta-analysis on the epidemiology of methylmalonic acidemia (MMA) with a focus on MMA caused by methylmalonyl-CoA mutase (mut) deficiency. Orphanet. J. Rare Dis..

[12] Shchelochkov O.A., Carrillo N., Venditti C., Adam M.P., Ardinger H.H., Pagon R.A., Wallace S.E., Bean L.J.H., Stephens K., Amemiya A. (1993). Propionic Acidemia. GeneReviews^®^.

[13] Summar M.L., Koelker S., Freedenberg D., Le Mons C., Haberle J., Lee H.S., Kirmse B., European Registry and Network for Intoxication Type Metabolic Diseases (E-IMD), Members of the Urea Cycle Disorders Consortium (UCDC) (2013). The incidence of urea cycle disorders. Mol. Genet. Metab..

[14] Hediger N., Landolt M.A., Diez-Fernandez C., Huemer M., Häberle J. (2018). The impact of ammonia levels and dialysis on outcome in 202 patients with neonatal onset urea cycle disorders. J. Inherit. Metab. Dis..

[15] Brassier A., Gobin S., Arnoux J.B., Valayannopoulos V., Habarou F., Kossorotoff M., Servais A., Barbier V., Dubois S., Touati G. (2015). Long-term outcomes in Ornithine Transcarbamylase deficiency: A series of 90 patients. Orphanet. J. Rare Dis..

[16] Martín-Hernández E., Aldámiz-Echevarría L., Castejón-Ponce E., Pedrón-Giner C., Couce M.L., Serrano-Nieto J., Pintos-Morell G., Bélanger-Quintana A., Martínez-Pardo M., García-Silva M.T. (2014). Urea cycle disorders in Spain: An observational, cross-sectional and multicentric study of 104 cases. Orphanet. J. Rare Dis..

[17] Posset R., Garcia-Cazorla A., Valayannopoulos V., Teles E.L., Dionisi-Vici C., Brassier A., Burlina A.B., Burgard P., Cortes-Saladelafont E., Dobbelaere D. (2016). Age at disease onset and peak ammonium level rather than interventional variables predict the neurological outcome in urea cycle disorders. J. Inherit. Metab. Dis..

[18] Nassogne M.C., Héron B., Touati G., Rabier D., Saudubray J.M. (2005). Urea cycle defects: Management and outcome. J. Inherit. Metab. Dis..

[19] Kölker S., Garcia-Cazorla A., Cazorla A.G., Valayannopoulos V., Lund A.M., Burlina A.B., Sykut-Cegielska J., Wijburg F.A., Teles E.L., Zeman J. (2015). The phenotypic spectrum of organic acidurias and urea cycle disorders. Part 1: The initial presentation. J. Inherit. Metab. Dis..

[20] Kido J., Matsumoto S., Mitsubuchi H., Endo F., Nakamura K. (2018). Early liver transplantation in neonatal-onset and moderate urea cycle disorders may lead to normal neurodevelopment. Metab. Brain. Dis..

[21] Enns G.M., Berry S.A., Berry G.T., Rhead W.J., Brusilow S.W., Hamosh A. (2007). Survival after treatment with phenylacetate and benzoate for urea-cycle disorders. N. Engl. J. Med..

[22] Kido J., Nakamura K., Mitsubuchi H., Ohura T., Takayanagi M., Matsuo M., Yoshino M., Shigematsu Y., Yorifuji T., Kasahara M. (2012). Long-term outcome and intervention of urea cycle disorders in Japan. J. Inherit. Metab. Dis..

[23] Kim I.K., Niemi A.K., Krueger C., Bonham C.A., Concepcion W., Cowan T.M., Enns G.M., Esquivel C.O. (2013). Liver transplantation for urea cycle disorders in pediatric patients: A single-center experience. Pediatr. Transplant..

[24] Morioka D., Kasahara M., Takada Y., Shirouzu Y., Taira K., Sakamoto S., Uryuhara K., Egawa H., Shimada H., Tanaka K. (2005). Current role of liver transplantation for the treatment of urea cycle disorders: A review of the worldwide English literature and 13 cases at Kyoto University. Liver Transplant..

[25] Husson M.C., Schiff M., Fouilhoux A., Cano A., Dobbelaere D., Brassier A., Mention K., Arnoux J.B., Feillet F., Chabrol B. (2016). Efficacy and safety of i.v. sodium benzoate in urea cycle disorders: A multicentre retrospective study. Orphanet. J. Rare Dis..

[26] Unsinn C., Das A., Valayannopoulos V., Thimm E., Beblo S., Burlina A., Konstantopoulou V., Mayorandan S., de Lonlay P., Rennecke J. (2016). Clinical course of 63 patients with neonatal onset urea cycle disorders in the years 2001–2013. Orphanet. J. Rare Dis..

[27] Posset R., Garbade S.F., Boy N., Burlina A.B., Dionisi-Vici C., Dobbelaere D., Garcia-Cazorla A., de Lonlay P., Teles E.L., Vara R. (2019). Transatlantic combined and comparative data analysis of 1095 patients with urea cycle disorders-A successful strategy for clinical research of rare diseases. J. Inherit. Metab. Dis..

[28] Mew N.A., Simpson K.L., Gropman A.L., Lanpher B.C., Chapman K.A., Summar M.L. (2003). Urea Cycle Disorders Overview. GeneReviews^®^.

[29] Bijarnia-Mahay S., Häberle J., Jalan A.B., Puri R.D., Kohli S., Kudalkar K., Rüfenacht V., Gupta D., Maurya D., Verma J. (2018). Urea cycle disorders in India: Clinical course, biochemical and genetic investigations, and prenatal testing. Orphanet. J. Rare Dis..

[30] Häberle J., Boddaert N., Burlina A., Chakrapani A., Dixon M., Huemer M., Karall D., Martinelli D., Crespo P.S., Santer R. (2012). Suggested guidelines for the diagnosis and management of urea cycle disorders. Orphanet. J. Rare Dis..

[31] Uchino T., Endo F., Matsuda I. (1998). Neurodevelopmental outcome of long-term therapy of urea cycle disorders in Japan. J. Inherit. Metab. Dis..

[32] Posset R., Gropman A.L., Nagamani S.C.S., Burrage L.C., Bedoyan J.K., Wong D., Berry G.T., Baumgartner M.R., Yudkoff M., Zielonka M. (2019). Impact of Diagnosis and Therapy on Cognitive Function in Urea Cycle Disorders. Ann. Neurol..

[33] Association of Public Health Laboratories NewSTEPs Screened Conditions Report. https://www.newsteps.org/data-resources/reports/screened-conditions-report.

[34] Trinh M.U., Blake J., Harrison J.R., Gerace R., Ranieri E., Fletcher J.M., Johnson D.W. (2003). Quantification of glutamine in dried blood spots and plasma by tandem mass spectrometry for the biochemical diagnosis and monitoring of ornithine transcarbamylase deficiency. Clin. Chem..

[35] Cavicchi C., Malvagia S., la Marca G., Gasperini S., Donati M.A., Zammarchi E., Guerrini R., Morrone A., Pasquini E. (2009). Hypocitrullinemia in expanded newborn screening by LC-MS/MS is not a reliable marker for ornithine transcarbamylase deficiency. J. Pharm. Biomed. Anal..

[36] Janzen N., Terhardt M., Sander S., Demirkol M., Gökçay G., Peter M., Lücke T., Sander J., Das A.M. (2014). Towards newborn screening for ornithine transcarbamylase deficiency: Fast non-chromatographic orotic acid quantification from dried blood spots by tandem mass spectrometry. Clin. Chim. Acta.

[37] Held P.K., Haynes C.A., De Jesús V.R., Baker M.W. (2014). Development of an assay to simultaneously measure orotic acid, amino acids, and acylcarnitines in dried blood spots. Clin. Chim. Acta.

[38] McHugh D., Cameron C.A., Abdenur J.E., Abdulrahman M., Adair O., Al Nuaimi S.A., Åhlman H., Allen J.J., Antonozzi I., Archer S. (2011). Clinical validation of cutoff target ranges in newborn screening of metabolic disorders by tandem mass spectrometry: A worldwide collaborative project. Genet. Med..

[39] Merritt J.L., Brody L.L., Pino G., Rinaldo P. (2018). Newborn screening for proximal urea cycle disorders: Current evidence supporting recommendations for newborn screening. Mol. Genet. Metab..

[40] Nettesheim S., Kölker S., Karall D., Häberle J., Posset R., Hoffmann G.F., Heinrich B., Gleich F., Garbade S.F., Arbeitsgemeinschaft für Pädiatrische Stoffwechselstörungen (APS) (2017). Incidence, disease onset and short-term outcome in urea cycle disorders -cross-border surveillance in Germany, Austria and Switzerland. Orphanet. J. Rare Dis..

[41] Lim J.S., Tan E.S., John C.M., Poh S., Yeo S.J., Ang J.S., Adakalaisamy P., Rozalli R.A., Hart C., Tan E.T. (2014). Inborn Error of Metabolism (IEM) screening in Singapore by electrospray ionization-tandem mass spectrometry (ESI/MS/MS): An 8 year journey from pilot to current program. Mol. Genet. Metab..

[42] Grosse S.D., Boyle C.A., Kenneson A., Khoury M.J., Wilfond B.S. (2006). From public health emergency to public health service: The implications of evolving criteria for newborn screening panels. Pediatrics.

[43] American College of Medical Genetics Newborn Screening Expert Group (2006). Newborn screening: Toward a uniform screening panel and system—Executive summary. Pediatrics.

[44] Hewlett J., Waisbren S.E. (2006). A review of the psychosocial effects of false-positive results on parents and current communication practices in newborn screening. J. Inherit. Metab. Dis..

