# Supplementary material for: Considering Proximal Urea Cycle Disorders in Expanded Newborn Screening
Source: Int J Neonatal Screen. 2020 Oct 8;6(4):77. doi: 10.3390/ijns6040077 (PMC7712149; doi:10.3390/ijns6040077)
Supplement: Supplementary file 1 [file IJNS-06-00077-s001.pdf]

Supplemental Materials:

Figure S1. Selection criteria for articles included in the study

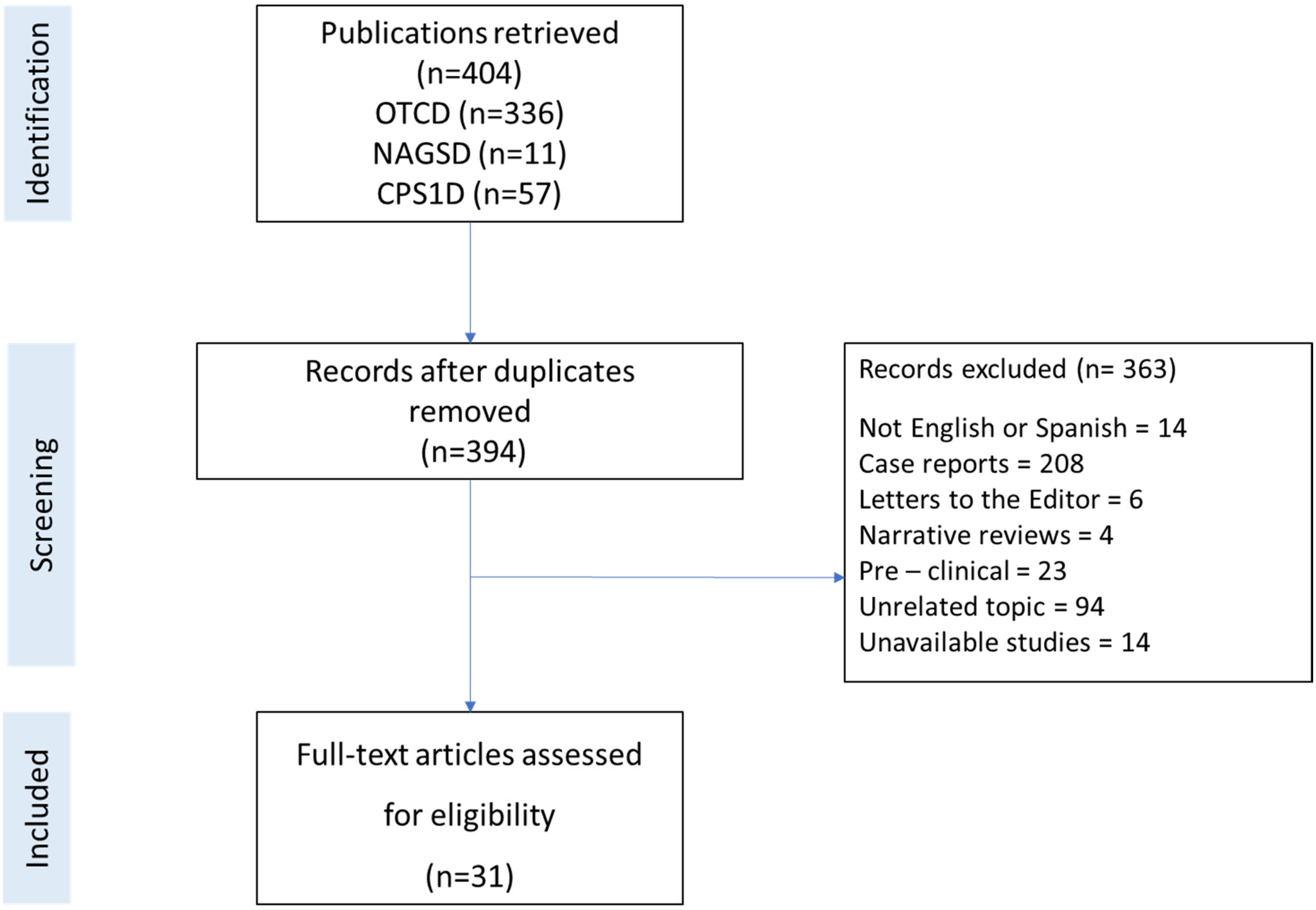

Table S1. Prisma Checklist (http://prisma-statement.org/)

Section/topic	#	Checklist item	Reported on page #
**TITLE**			
Title	1	Identify the report as a systematic review, meta-analysis, or both.	1
**ABSTRACT**			
Structured summary	2	Provide a structured summary including, as applicable: background; objectives; data sources; study eligibility criteria, participants, and interventions; study appraisal and synthesis methods; results; limitations; conclusions and implications of key findings; systematic review registration number.	1
**INTRODUCTION**			
Rationale	3	Describe the rationale for the review in the context of what is already known.	2
Objectives	4	Provide an explicit statement of questions being addressed with reference to participants, interventions, comparisons, outcomes, and study design (PICOS).	2
**METHODS**			
Protocol and registration	5	Indicate if a review protocol exists, if and where it can be accessed (e.g., Web address), and, if available, provide registration information including registration number.	NA
Eligibility criteria	6	Specify study characteristics (e.g., PICOS, length of follow-up) and report characteristics (e.g., years considered, language, publication status) used as criteria for eligibility, giving rationale.	2
Information sources	7	Describe all information sources (e.g., databases with dates of coverage, contact with study authors to identify additional studies) in the search and date last searched.	2
Search	8	Present full electronic search strategy for at least one database, including any limits used, such that it could be repeated.	2
Study selection	9	State the process for selecting studies (i.e., screening, eligibility, included in systematic review, and, if applicable, included in the meta-analysis).	2
Data collection process	10	Describe method of data extraction from reports (e.g., piloted forms, independently, in duplicate) and any processes for obtaining and confirming data from investigators.	2
Data items	11	List and define all variables for which data were sought (e.g., PICOS, funding sources) and any assumptions and simplifications made.	NA
Risk of bias in individual studies	12	Describe methods used for assessing risk of bias of individual studies (including specification of whether this was done at the study or outcome level), and how this information is to be used in	NA

		any data synthesis.	
Summary measures	13	State the principal summary measures (e.g., risk ratio, difference in means).	NA
Synthesis of results	14	Describe the methods of handling data and combining results of studies, if done, including measures of consistency (e.g., I^2) for each meta-analysis.	NA

Table S2. Summary of Literature Included in Systematic Review

Study	Summary
Uchino et al 1998, (Japan) [31]	This study reports a cohort of 92 patients with neonatal onset and 116 patients with LO UCD from 1978–1995. OTC was the most common disorder (n=144, EO=45, LO=99). The 5-year survival rate for patients with LO – OTCD was 42% (42/99). Peak ammonia level <180 µmol/L was not linked to developmental delay in the long term.
Mew et al 2003, (USA) [22]	Urea Cycle Disorders Overview
Trinh et al 2003, (Australia) [33]	This study demonstrates the instability of glutamine and, to a lesser extent, glutamic acid in dried blood spots under typical storage conditions.
Nassogne et al 2005, (Belgium-France) [18]	This study presents 217 patients. 75.5% (164) had a PUCD. Among those with PUCDs, 49% (81) had EO and (51%) 83 had LO. Males with EO OTCD have the highest mortality rate (60-70%). Among patients with LO OTCD, 34% (28/32) died. Regarding developmental delay, it was present in 37% (31/82) of them. 70% (22/31) of patients with LO OTCD and developmental delay were females and 64% (20/31) of them had a moderate neurological deficit.
Hewlett et al 2006, (USA) [41]	"This is a literature review about the negative impact of false positive newborn screening results on parents. Parental stress and anxiety can be reduced with improved education and communication."
Enns et al 2007, (United States) [26]	Over 25 years, 299 patients with UCDs were treated with sodium phenylacetate and benzoate. Overall survival was 84% (250 of 299 patients). 96% of the patients survived episodes of hyperammonemia (1132 of 1181 episodes). Patients over 30 days of age were more likely than neonates to survive an episode (98% vs. 73%, P<0.001). Patients 12 or more years of age, who had 437 episodes, were more likely than all younger patients to survive. 81% of patients who were comatose at admission survived. Patients less than 30 days of age with a peak ammonium level >1000 µmol/L were least likely to survive a hyperammonemic episode.<br>Among those patients with EO OTCD (n=43), 60.5% (n=26) survived the first episode and 53% (n=23) survived all known episodes. Mortality for all known episodes was 47%.<br>There was a 79% decrease from baseline after treatment with sodium benzoate and arginine.<br>Ammonia change in subgroups: decreased EO: from 334 to 364 µmol/L; survivors EO, from 374 to 24 µmol/L.

Summar et al 2008, (USA) [8]	A large longitudinal interventional study of patients with a urea cycle disorder (UCD) in hyperammonemic crisis (1982–2003).<br>For patients with AS or CPS-I deficiencies and males with OTC deficiency, approximately 70% of episodes. Occurred between the age of 31 days and 12 years. For females with OTC deficiency, 92% of episodes occurred at age >2 years.<br>The treatment for hyperammonemic crises caused by UCDs was an intravenous combination of nitrogen scavenging drugs. 34% out of 260 patients presented within the first 30 days of life and the mortality rate was 32%. Survival was better among patients with CPS1D and females with OTCD.
Cavicchi et al 2009, (Italy) [34]	6/48000 newborns showed decreased citrulline levels, which turned out to be false positive in 5/6 patients, because of prematurity and intestinal malrotation.<br>The 6th case was a positive OTCD case, identified via molecular genotyping and a family history of transaminitis. Citrulline was given for treatment. A follow up at 3 years of age showed a healthy infant with a normal development.
McHugh et al 2011, (USA) [37]	Cumulative percentiles of amino acids and acylcarnitines in dried blood spots of approximately 25–30 million normal newborns and 10,742 deidentified true positive cases are compared to assign clinical significance. The cutoff target ranges of analytes and ratios are then defined as the interval between selected percentiles (either 1st or 99th percentiles) of the two populations. When overlaps occur, adjustments are made to maximize. Sensitivity and specificity taking all available factors into consideration. The overall proportion of cutoff values within the respective target range was 42%. For CPS1D and OTCD, they used markers such as citrulline to arginine, citrulline to phenylalanine, glutamine/citrulline, glutamic acid/citrulline, and methionine/citrulline ratios.
Kido et al 2012, (Japan) [29]	In Japan, the prevalence of UCDs is 1/50,000 and 177 patients with UCDs between 1999–2009 (OTCD 116/177). Among OTCD (n= 116), EO=24% (28/116), LO 68% (80/116), unknown n=8. Among OTCD males (n=57): 37% had neonatal onset, 53% late onset, and unknown 10%. Among OTCD females (n=59): 12% had neonatal onset, 85% had late onset and 3% were unknown. The 5-year survival rate of patients with OTCD was 86% (21/28) for those with neonatal-onset, and 92% (74/80) for those with LO. The 10-year survival rate for the three types of OTCD, except for female neonate-onset OTCD, was more than 80%. The survival rate at 20 years of age for patients with LO OTCD was 89.4% in males and 83.8% in females. 23% (27/116) patients with OTCD underwent liver transplant.
Häberle et al 2012, (Austria) [24]	Guideline for the diagnosis and management of urea cycle disorders.

Häberle et al 2019, (International) [6]	Guideline for the diagnosis and management of urea cycle disorders.
Summar et al 2013, (International) [13]	This is a longitudinal study that uses US and European data to find out prevalence of UCDS: NAGS <1:2,000,000; CPS1D 1:1,300,000; OTCD 1:56,500.
Kim et al 2013, (USA) [28]	This study reports 23 children affected with UCDs who underwent liver transplant.<br>Fifteen (65%) patients received a whole-liver graft, seven patients (30%) received a reduced size graft, and one patient received a living donor graft. Mean 74% (17/23) had an EO UCD[15], 5 had CPSD, 8 had OTCD (4 females). For the EO PUCDs, age of transplant varied (CPSD 6-203 weeks, OTCD males 3-6 weeks, and OTCD females 50-119 weeks).<br>Patient survival at one and 5 years were 100% each time. After transplant ammonia levels (28 µmol/L) decreased compared to before transplant levels (772 µmol/L, range: 178–2969 µmol/L). There were no episodes of hyperammonemia post-transplant.
Lim et al 2014, (Singapore) [36]	This study shares the experience of 8 years of tandem mass spectrometry in Singapore. The incidence rate of OTCD was 1:177,000 and the PPV was 20%.
Martin-Hernandez et al 2014 (Spain) [16]	This study reports 104 Spanish patients reported from 2012–2013 (OTCD=67, CPS1D=2 and NAGSD=1). Among the 67 patients with OTCD: 9 had EO (13%), 52 LO (78%) and 6 (9%) were asymptomatic.<br>Of EO OTCD: 73% (7/9) were males, 22% (2/9) were females. Among LO OTCD: 29% (15/52) male and female 71% (37/52). In the asymptomatic group, 4 were males.<br>By the time of their patient report, mortality was present in 40% (2/5) EO OTCD. No deaths were reported for LO OTCD. Neurological damage affected 48% (20/42) LO OTCD (see Table 4 in Martin-Hernandez's article) and it was not reported for EO OTCD. It was present in 2/2 patients with CPS1D and 1/2 with NAGSD.<br>Most of them have a protein restrictive diet.

Brassier et al 2015, (France) [15]	OTCD only (n=90) from France (1971–2011). EO 27/90 and LO 63/90. Overall, 48/90 (53%) were male and 42/90 (47%) were female. For EO OTCD: median age of onset of 2 days old, 5/27 (19%) were females, 22/27 (81%) were males. For EO OTCD, the mortality rate was 74% (20/27). Furthermore, with 60% of those with EO died within the 1st week (See Figure 1). Among those EO survivors, they had 1 decompensation/year until the end of this study (mean per patient 6.2). For LO OTCD, 58% (52/90) presented 1 month to 16 years of age, with a median of 1.3 years of age; 31/52 were female, 21/52 were male were women and 13.4% (7/52) of them died. Regarding the neurological outcome, among the EO patients, 71% (5/7) of the survivors had a mean IQ=90 (67-91), 3/5 had a normal schooling and 2/5 had remedial schooling. Among patients with LO onset ages 1 month–16 years, 20/52 patients had a median IQ =92 (55-103). Among 38/52 with a complete documentation about IQ and schooling: 71% (27/38) had normal schooling, 29% (5/38) had either no or remedial schooling had no schooling due to severe disability, and 15.7% (6/38) had remedial schooling).<br>86% of the participants had a deleterious mutation.
Kölker et al 2015 (Germany) [19]	This study describes 795 patients with OAD (n= 452) and UCD (n=343, PUCDs 219, DUCDs 124, OTCD n=196). Among those with PUCDs, 17% (37) had EO, 53% (114) had LO and 30% (68) were asymptomatic.<br>The majority (n=463) presented with acute metabolic crisis during or after the newborn period. Neonatal onset was more frequent in CPS1D and low in OTCD. Hyperammonemia was more severe in metabolic crises during than after the newborn period. There was a delay in diagnosis for symptomatic patients with UCDs without metabolic crises presented with psychiatric disorders. Late onset was more frequent in females, from which 6 patients had a late onset acute crisis. Impaired consciousness was the most frequent symptom among patients with neonatal and late onset. However, duration was longer in those with late onset than early onset.
Morioka et al 2015, (Japan) [30]	This is a cohort of 21 patients with noncirrhotic inheritable metabolic liver disease (1999–2003). 6/21 of them had OTCD. Overall, survival rate was 85.7% at both 1- and 5-years post-transplant. At 5 years follow-up, overall, there was an 83.3% and 78% survival rate for the APOLT (auxiliary partially orthotopic liver transplantation) and non-APOLT group, with no statistical significance. There was an 83% (5/6) survival rate for patients with OTCD at 5 years follow-up, with one non transplant related death (accident).
Husson et al 2016 (France) [27]	This is a 10–year retrospective cohort study reporting the efficacy and safety of sodium benzoate among 61 patients with UCDs. This study shows that sodium benzoate was effective in decreasing ammonium levels ≤100 μmol/L in 92.8% of the episodes.

Posset et al 2016, (Germany) [17]	Total number of UCD patients: 456. PUCD: 255 OTCD (OTCDm=109, OTCDf=146), 21 CPS1D, 9 NAGSD. DUCDs: ASSD 87, ASLD 61, ARG1D 12, and HHH 11.<br>The overall median age at diagnosis was lower in the NBS group (median: 12 days; IR: 6-14 days) than<br>in the LO group of patients identified after manifestation of first symptoms (median: 730 days; IR: 365-2160 days). In contrast, age at diagnosis was similar or even lower in the EO group (median: 4 days; IR: 3-9 days) than in the NBS group (median: 12 days; IR: 6-14 days).<br>Overall, 70% (day 6) and 65% (day 12) of UCD patients identified by NBS and selective metabolic investigation, respectively, have remained asymptomatic and, theoretically, the manifestation of first symptoms could have been prevented by NBS in this group.<br>Overall, ORs showed a trend towards lower odds for movement disorder in the NBS and early diagnosis groups compared to the selective metabolic investigation group.
Unsinn et al 2016 (Switzerland) [25]	This study reports a cohort of 63 patients with EO UCDs during the period 2001–2013. 36% (23/63) of the patients had OTCD. Patients with an ammonia ≥500 µmol/L developed encephalopathy and required dialysis. 28.5% of these patients died. Mortality among patients with EO OTCD was present in 43% (10/23) of them.
Nettesheim et al 2017, (Europe) [35]	This is a cross-border surveillance of European patients <16 years old diagnosed with a UCDs (2012–2015). It describes 50 patients (OTCDm n=17, OTCDf n=5; CPS1D n=8; ASSD n=10; ASLD n=7; ARGD n=1; Citrin D n=1; HHH n=1). Patients with CPS1D, OTCD males and females had hyperammonemic encephalopathy as the initial presentation. 28/50 had neonatal onset. In this cohort, 11 patients were asymptomatic and diagnosed through NBS (10/11, OTCD 1, ASSD 4, ASLD 3, ARG1D 1 and Citrin D 1) and a family risk assessment (1/11, OTCD 1). Only 1 patient identified by NBS developed an early onset disease on the 5th day of life, however condition and the outcome for this case are not described in the study.

Bijarnia-Mahay et al 2018, (India) [23]	This study reports 123 patients from India 2001–2007. For the diagnosis, MS/MS, U/HPLC and GC-MS were used. ASS1D n= 61, OTCD n=26, CPS1D n=3, NAGSD n=2. Family history of OTCD was present in 50% of the patients with OTCD.<br>58% (72/123) of overall patients presented within the first 30 days of life and 88% of them had the initial presentation day at ≤7 of life. Overall, the mortality rate was 64% (70/110), and 70% (28/40) of the survivors developed a disability.<br>Regarding OTCD patients, 11/26 had an EO presentation with mortality in 11/26 and developmental delay in 7/26 patients. A molecular mutation was reported in 82% (18/22) of the OTCD patients who had sequencing, in 3/3 CPSD and 2/2 NAGSD patients. No molecular diagnosis was present in 18% (4/22) OTCD patients, with the remaining 4/26 with no molecular testing.
Hediger et al 2018, (Switzerland) [14]	This is a systematic review about 202 patients with EO UCDs. Median age at onset was three days old and mean ammonia that triggered start of dialysis was 1199 µmol/L. 71% of all patients received any form of dialysis. Total mortality was 25% and only 20% of all patients had a normal outcome. Mortality was present in 33% of the patients who were affected with a PUCD. Mortality affected 1/9 patients with NAGSD, 14/43 patients with CPSD and 24/66 patients with OTCD. The percentage of deceased patients decreased continuously over time from 50% (n=10) in publications between 1971 and 1990 to 20.8% (n=16) reported between 2011 and 2016.
Kido et al 2018, (Japan) [20]	This study reports a cohort of 177 Japanese UCD patients that received healthcare from January 1999–March 2009. The most common condition was OTCD (n=116). This study suggests peak ammonia levels are linked to poorer neurodevelopmental, thus it recommends liver transplantation as early as possible in EO cases even when mean ammonia concentration is <300 µmol/L.
Merritt et al 2018, (USA) [38]	This study reviews 11 cases of UCDs and proposes a new tool for screening of PUCDs. This tool uses a combination of analytes that are already measured with current tandem mass spectrometry technologies. It detected 12 known EO and LO PUCD patients except for one asymptomatic LO OTC patient and a second patient with an incomplete set of analytes. Both had normal citrulline levels.
Posset et al 2019, (Germany) [21]	Diagnostic delays can be significant—ranging from 1 day up to 1134 days for patients with EO OTCD and EO CPS1D, even to extremely large times of 3652 days in a child with NAGSD—when depending upon development of disease symptoms to initially identify disease.
Posset et al 2019, (Germany) [32]	51 individuals had a liver transplant (CPS1D n=6; OTCDm n=18; OTCDf n=5; ASSD n=12; ASLD n=10) and 303 received conservative treatment. Comparative ANOVA was used to compare 45 transplanted individuals (CPS1D n=6; OTCDm n=18; OTCDf n=5; ASSD n=8;

	ASLD n=8) to those who did not receive a transplant. Post-transplant cognitive scores performed better in LO individuals ($p=0.026$, ANOVA) and those who received a timely transplantation independent from the disease onset ($p=0.087$, ANOVA). There was no interaction between disease onset and time of transplantation. Individuals with ASSD (n=15) and ASLD (n=23) identified by NBS had normal mean cognitive score that was higher than in individuals diagnosed after the manifestation of first symptoms (each $p<0.001$).
Janzen et al 2014, (Germany) [35]	This study provides a new method that measures orotic acid via fast non-chromatographic quantification in dried blood spots using MS/MS. The method provides a clear distinction of normal newborn samples compared to those of affected newborns with OTCD (average; range: 0.914; 0.28–3.73 µmol/L vs 144.1; 89.7-211.1 µmol/L), with similar reported values were observed when categorizing for gestational age and sex.
Held et al 2014, (USA) [36]	Orotic acid was extracted from 24-96hr DBS and analyzed using flow-injection analysis tandem mass spectrometry (FIA–MS/MS) with negative-mode ionization, requiring <2 min/sample run time. This method was then multiplexed into a conventional newborn screening assay for analysis of amino acids, acylcarnitines, and orotic acid. In the stand-alone method, the measurement of orotic acid in samples of healthy newborns were on average 1.20 µmol/l (1st–99th percentile: 0.72–1.84 µmol/l). For the combined method, the concentration was on average 1.29 µmol/l (1st–99th percentile 0.50–2.85 µmol/l). The orotic acid concentration in 2 males with OTCD were 11 and 21.80 uM. The article doesn't specify whether these cases had an early presentation.

EO Early onset, LO Late onset, NAGSD N-acetylglutamate synthetase deficiency, CPS1D carbamoyl phosphate synthetase 1 deficiency, OTCD ornithine transcarbamylase deficiency, ASSD argininosuccinate synthetase deficiency, ASLD argininosuccinate lyase deficiency, LT liver transplant, SB sodium benzoate, SP sodium phenylacetate, SPB sodium phenylbutyrate, HF hemofiltration, HD hemodialysis, PUCD proximal urea cycle disorders, DUCD distal urea cycle disorders.
